# Lactate-driven macrophage polarization in the inflammatory microenvironment alleviates intestinal inflammation

**DOI:** 10.3389/fimmu.2022.1013686

**Published:** 2022-10-18

**Authors:** Hai-Cun Zhou, Wen-Wen Yu, Xin-Yan Yan, Xiao-Qin Liang, Xiu-Feng Ma, Jian-Ping Long, Xiao-Yan Du, Hong-Yan Mao, Hong-Bin Liu

**Affiliations:** ^1^ The Second Clinical Medical College, Lanzhou University, Lanzhou, China; ^2^ Department of Breast Surgery, Gansu Maternal and Child Health Care Hospital, Lanzhou, Gansu Province, China; ^3^ Key Laboratory of Stem Cells and Gene Drugs of Gansu Province, The 940th Hospital of Joint Logistics Support Force of Chinese People’s Liberation Army, Lanzhou, China

**Keywords:** lactic acid, inflammation, colitis, macrophage polarization, inflammatory microenvironment

## Abstract

**Background:**

Lactate has long been considered an intermediate by-product of glucose metabolism. However, in recent years, accumulating evidence reveals that lactate has unique biological activities. In previous studies, lactate signaling was shown to inhibit inflammation. Furthermore, *in vitro* experiments have shown that lactate can promote the transformation of pro-inflammatory macrophages into anti-inflammatory macrophages. However, no *in vivo* studies have shown whether lactate can alleviate inflammation.

**Methods:**

RAW 264.7 macrophages were stimulated by LPS to induce an M1 phenotype, and cultured with low and high concentrations of lactate. The cells were then observed for phenotypic transformations and expression of inflammatory mediators and surface markers. The expression of inflammatory factors was also analyzed in the cell-free supernatant fraction. Further, a mouse model of DSS-induced colitis was established and treated with lactate. Colonic tissue injury was monitored by histopathological examinations.

**Results:**

The *in vitro* experiments showed that lactate promoted the transformation of activated macrophages to M2 phenotype and decreased the expression of TLR4-mediated NF-κB signaling proteins and inflammatory factors. In the DSS-induced colitis mouse model, lactate promoted the phenotypic transformation of macrophages in colonic tissue, reduced inflammation and organ damage, inhibited the activation of TLR4/NF-κB signaling pathway, decreased the serum levels of pro-inflammatory factors, increased the expression of anti-inflammatory factors, promoted the repair of the intestinal mucosal barrier and reduced the severity of colitis.

**Conclusions:**

Lactate inhibits the TLR/NF-κB signaling pathway and the production of pro-inflammatory factors by promoting polarization of macrophages. In addition, lactate promotesthe repair of the intestinal mucosal barrier and protects intestinal tissue in inflammation. Furthermore, lactate is relatively safe. Therefore, lactate is a promising and effective drug for treating inflammation through immunometabolism regulation.

## Introduction

Inflammatory bowel diseases (IBD) are a group of chronic inflammatory diseases of the digestive tract and include Crohn’s disease (CD) and ulcerative colitis (UC). Inflammatory bowel diseases affect people of all ages (1). The etiology of IBD remains unknown. However, it is thought to be due to a combination of genetic, microbial, immunological and environmental factors. These factors lead to an imbalanced immune response characterized by pro-inflammatory phenotype with elevated tissue concentrations of various cytokines, including tumor necrosis factor (TNF), interleukin-6 (IL-6), and interferon-γ (IFNγ) (2). The immune response leads to local mucosal damage, characterized by loss of tight junction proteins and damage to the intestinal epithelial cells and the villi (3).

Several studies have shown that macrophage polarization is closely related to the occurrence and development of various inflammatory diseases, such as cardiovascular diseases, autoimmune diseases, obesity, and diabetes (4, 5). Macrophages can switch their phenotypes from M1 to M2 and vice versa. The two phenotypes play different functions. In the early stages of inflammation, the M1-type macrophages are recruited and lead to the activation of the adaptive immune cells. The released pro-inflammatory cytokines induce Th1 and Th17 responses to eliminate the offending insult. However, these responses may cause damage to normal tissues. During the resolution of inflammation, the M1-phenotype is transformed into an anti-inflammatory M2-phenotype, which induces the Th2 response and participates in tissue healing and repair (6). An increasing number of studies studies have explored the regulation of a balance between pro-inflammatory and anti-inflammatory responses as a novel therapeutic strategy for anti-inflammatory diseases (7–9).

Lactate has long been regarded as a by-product of metabolism rather than a biologically active molecule. However, in recent years, lactate has been shown to be involved in signaling. In addition, studies have shown that lactate promotes inflammation (10–12). In recent years, accumulating evidence show that lactate is related to the metabolism and polarization of macrophage. During inflammation, lactate is a regulator of macrophage metabolism and can act as a negative feedback signal to prevent excessive inflammatory response (6). A previous study showed that lactate stimulated resident macrophages in tumors to polarize into M2-type, which played an important role in immune suppression and cancer progression (13). Colegio et al. (14) demonstrated that the effect of lactate in promoting M2-like polarization of TAMs was mediated by HIF-1α. In 2019, Zhang et al. revealed that lactate promoted histone lactylation in macrophages, an epigenetic modification that leads to the transcription of anti-inflammatory genes (15). Lactate is an important signaling molecule for regulating immune responses. Therefore, targeting lactate offers a promising therapeutic strategy in inflammatory diseases.

So far, no studies have investigated whether lactate can alleviate inflammation *in vivo* by promoting macrophage polarization. Therefore, this study investigated the *in vivo* and *in vitro* effects of lactate in inflammatory bowel diseases.

## Materials and methods

### Materials

LPS (S1735) was purchased from Beyotime Biotechnology. Dextran sulfate sodium salt (DSS, MW 35000 -45000, CAS NO.9011-18-1) was purchased from MedChemExpress. The Hoechst 33342 (10ug/ml, C0030), reactive oxygen species assay kit (CA1410), lactate content detection kit (BC2235), and BCA protein assay kit (PC0020) were purchased from Solarbio Life Sciences (Beijing, China). Cell counting kit-8 (CCK-8) was purchased from Yeasen Biotech Co. (40203). The Mouse TNF-α ELISA kit was purchased from NeoBioscience (EMC102a, Shenzhen, China). Mouse IL-1β (JL18442), TGF-β (JL13959), and IL-10 (JL20242) ELISA kits were purchased from Jianglai Biotechnology Co., Ltd. (Shanghai, China). The anti­CD86 antibodies (NBP2-6882A) were purchased from Novus Bio. The anti­CD206 antibodies (ab64693), anti­Arg1 antibodies (ab233548), anti­iNOS antibodies (ab178945), anti-ZO-1 tight junction protein antibodies (ab216880), anti-occludin antibodies (ab216327), anti-claudin-1 antibodies (ab15098), and anti-β actin antibodies (ab8227) were purchased from Abcam. Rabbit anti-p65 antibodies (FNab09875) and rabbit anti-TLR4 antibodies (FNab08727) were purchased from Fine Biotech Co. Ltd (Wuhan, China). Goat anti-rabbit IgG(H+L) FITC conjugate (SA00003-2) and goat anti-mouse IgG(H+L) rhodamine conjugate (SA00007-1) were purchased from Proteintech Group. The secondary antibodies [peroxidase-conjugated goat anti­mouse IgG (ZB2305) and peroxidase-conjugated goat anti­rabbit IgG (ZB2301)] were purchased from ZSGB-BIO (Beijing, China).

### Cell culture

Mouse macrophages (Raw264.7, FH0328) were purchased from Shanghai Fuheng Science & Technology Co., Ltd. The Raw264.7 cells were cultured in a high glucose Dulbecco’s Modified Eagle Medium (DMEM) containing 10% fetal bovine serum, 1% penicillin, and streptomycin. The cells were cultured in a humified incubator supplemented with 5% CO_2_ at 37°C. In addition, the cells were passaged three times a week.

### *In vitro* experiments for macrophage polarization

(i) Raw264.7 cells were inoculated onto six-well plates at a density of 4×10 (4) and cultured. Upon reaching a confluence of 70%, the cells were stimulated with 100ng/ml LPS for 24h to induce an M1 phenotype. The activated macrophages were cultured with low (10mM) and high (20mM) doses of lactate. Negative controls were set by culturing the activated macrophages with phosphate-buffered saline (PBS). The cells and cell-free supernatants were collected after 24 hours. (ii) Activated macrophages were cultured with lactate (15mM) and the cells, and cell-free supernatants were collected after 12h and 24h. The morphology of macrophages and the expression of surface markers, CD86 (red fluorescence) and CD206 (green fluorescence), associated with M1 and M2 macrophages, respectively, were observed under an inverted fluorescence microscope (OLYMPUS, 1X71+DP71). The expression markers CD86, CD206, iNOS, Arg1, and proteins associated with inflammatory TLR4/NF-κB signaling pathway were detected by Western blotting. Intracellular ROS levels were detected by fluorescence microscopy. Levels of the pro-inflammatory and anti-inflammatory factors in the supernatant were detected by ELISA.

### Immunofluorescence staining

RAW264.7 cells were inoculated in six-well plates to a density of 1×10 (5) and cultured. The medium was removed, and the cells were washed twice. After that, the cells were fixed in 4% paraformaldehyde at room temperature for 20min. Subsequently, the cells were washed thrice in pre-cooled PBS. Further, 1ml BSA (5mg/mL) was added, and the cells were incubated at 4°C for 1h. The primary antibodies (anti-CD86 antibodies and anti-CD206 antibodies) were then added to the cells and incubated overnight. After that, the macrophages were washed three times in PBS. Subsequently, the fluorescent-labeled secondary antibodies were added, and the cells were incubated in darkness for 2h at 4°C. After that, the cells were washed three times in pre-cooled PBS. Hoechst33342 was then added for nuclear staining. Fluorescence intensity was observed under an inverted fluorescence microscope (OLYMPUS, 1X71+DP71).

### Detection of intracellular ROS levels

RAW264.7 cells were inoculated in six-well plates at a density of 1×10 (5) and cultured. After that, the medium was removed, and the cells were washed twice. Subsequently, DCFHDA (1ul/ml) was added, and the cells were incubated at 37°C for 20min. After that, the cells were washed thrice with a serum-free medium. Fluorescence intensity was observed with an inverted fluorescence microscope (OLYMPUS, 1X71+DP71).

### Experimental animals

Male BALB/C mice, aged 8-12 weeks and weighing 22-24g, were purchased from the Animal Centre of the 940th Hospital of Joint Logistic Support Force of PLA. All animal studies were carried out in accordance with the recommendations of the Animal Protection Committee of the 940th Hospital of the Joint Logistic Support Force. All mice were housed undera specific pathogen free environment of standard humidity (52% ± 2%) and temperature (21 ± 2°C) conditions with 12 hr dark/light cycle. All mice were allowed *ad libitum* access to food and water. The mice were subjected to adaptation training for one week.

### Mouse model for colitis

Acute colitis in BALB/c mice was induced by adding 3% sodium dextran sulfate (DSS) to the drinking water which was provided to the animals for ten days. The water intake was monitored daily. The lactate solution (low and high doses:100mM/200mM) in PBS (200µL) was administered by transrectal perfusion through a polyurethane catheter (18 G), which was inserted 4cm near the anal edge of the colon. The mice were then held in a head-down position for 30s. The control group was provided with drinking water containing PBS. The drug was administered for the next day at a time within ten days. The mice were then monitored daily for body weight, mental state, activity levels, hair luster, appetite, and stool features. Further, the disease activity Index (DAI) was calculated. Each of the above parameters was scored on a scale of 0-4 with a maximum score of 12 (**Table 1**) (1, 16). After ten days, the mice were euthanized, and the colons were harvested for determination of the colon length, which was measured in centimeters. In addition, the serum was obtained for further analysis.

**Table 1:** Scoring system for Disease Activity Index (DAI).

Score	Weight loss	Stool consistency	Blood stool
0	no loss	normal	no blood
1	1-5%	loose stool	
2	5-10%	watery diarrhea	presence of blood
3	10-20%	slimy diarrhea, little blood	
4	>20%	severe watery diarrhea with blood	gross bleeding

### *In vivo* quantification of serum lactate levels

Quantitative determination of serum lactate was conducted using a lactate content detection kit (micro method). Healthy mice were administered with lactate solution (200mM) in PBS (200µL) by transrectal perfusion. After that, serum was collected at 3h, 6h, 12h and 24 hours to determine the lactate levels. The negative control group was treated with PBS. The serum lactate levels were determined using a multimode plate reader (Infinite M200 PRO) per the the kit manufacturer’s instructions.

### The villus length and the histological scoring

One centimeter of the distal colon was fixed in 10% formaldehyde followed by paraffin embedding. The colon tissue was then sectioned into 5μm thick sections dewaxed in xylene, dehydrated with gradient ethanol, and stained with hematoxylin for five minutes and with eosin for one minute. The colon sections were then observed under a microscope (Olympus, BX53, Japan). The villus length (μm) of the HE-stained sections was determined using Case Viewer 2.0 software (3DHISTECH Ltd.). Villus length was defined as the distance from the top of the villi to the muscularis of the intestinal wall. The gross morphological score for colon injury was evaluated based on the parameters shown in **Table 2** (1). The samples were evaluated by experienced pathologists who were blinded to each other.

**Table 2:** Scoring criteria of gross morphology of colon.

Score	Gross morphology
0	Normal gross morphology
1	Slightly thickened of colon wall but not congested
2	Moderately thickened of colon wall with congestion
3	Significantly thickened and stiff of colon wall with congestion
4	Significantly thickened and stiff of colon wall with congestion and adhesion

### Phenotypic transformation of macrophages *in vivo*

Colon tissues were fixed in formaldehyde, paraffin-embedded and sectioned. After fixation and permeabilization with 0.1% Triton X-100 (room temperature, 10 min), the tissues were sealed with normal goat serum (37°C, 1h) and incubated with anti-CD86 antibodies and anti-CD206 antibodies at 4°C overnight. After that, the tissues were incubated with the fluorescent secondary antibodies at room temperature for 1h, followed by incubation with Hoechst33342 for 15 min. The tissues were visualized under a fluorescence microscope (OLYMPUS,1X71+DP71).

### Detection of cytokines by ELISA

Blood samples from the mice and the medium supernatant were centrifuged at 3000 rpm for 10 min to obtain the serum. Levels of TNF-α, IL-10, IL-1β, and TGF-β in serum and the cell culture supernatant were determined using the ELISA kits. The results were read on a multimode plate reader (Infinite M200 PRO).

### Protein detection by western blotting

Cell and tissue proteins were extracted and quantified using a BCA protein assay kit. Similar quantities of protein samples were loaded onto an SDS-PAGE gel system. After electrophoresis, the proteins were transferred to a polyvinylidene fluoride (PVDF) membrane, which was then sealed with 5% skim milk powder at room temperature for 2 h. After that, the membranes were incubated overnight at 4°C with the following primary antibodies: anti-mannose receptor antibodies, anti-CD86 antibodies, anti-iNOS antibodies, anti-arg1 antibodies, anti-NF-κB P65 antibodies, anti-TLR4 antibodies, and anti-β-actin antibodies. The membrane was then washed three times with TBST containing 0.1% Tween-20 and incubated at room temperature for 2 hours with peroxidase-conjugated secondary antibodies. The separated bands were visualized using enhanced chemiluminescence kits and gel imaging system (4600, Tanon, China).

### Detection of protein expression by immunohistochemistry

Immunohistochemistry (IHC) was used to detect the expression of tight junction proteins in the colon tissues. The colon tissue samples were paraffin-embedded, sectioned, baked, dewaxed, and dehydrated with graded ethanol. The colon tissues were treated with primary antibodies (anti-Occludin antibodies, anti-ZO-1 antibodies, and anti-Claudin-1 antibodies) at 37°C for 2 h. After that, the tissues were incubated with secondary antibodies at 37°C for 30 min. Finally, the tissues were stained with diaminobenzidine (DAB) and hematoxylin and observed under a microscope (Olympus, BX53, Japan).

### Cytotoxicity assay

The CCK8 method was used to assess *in vitro* cytotoxicity of lactate. RAW264.7 cell suspension was added to a 96-well plate (100μL/well). The plate was then incubated for 24 hours (37°C, 5% CO_2_). After that, the medium was discarded, and 100ul/well of different concentrations of lactate (0mM, 5mM, 10mM, 20mM, 40mM, 80mM, 160mM) were added to each of the wells. Further, the plate was further incubated for 24 hours after which the medium was removed. After that, the cells were washed twice and a new medium was added. Subsequently, 10ul CCK8 solution was added to each well and incubated for 1-4 hours. The absorbance at 450nm was measured using a multi-mode reader (Infinite M200 PRO). In addition, the cells were assessed for viability. *In vivo* assessment for lactate cytotoxicity was conducted by administering lactate solution (200mM) in PBS (200µL) by transrectal perfusion to the healthy mice once daily for five days. The weight of the mice was recorded daily. After end point, the mice were sacrificed and the heart, liver, lungs, kidney, and spleen) were harvested for H&E staining. Negative controls were obtained by administering the mice with PBS.

### Statistical analysis

GraphPad Prism 7.0 (GraphPad Software, USA) was used for statistical analysis and graphing. All data were expressed as mean ± standard deviation (SD). Differences between groups were determined using the student’s T-test (two-tailed), one-way analysis of variance (ANOVA), two-way ANOVA, or the Kruskal-Wallis H test. Further, multiple posthoc comparisons were performed using the Tukey posthoc test with ANOVA to determine differences between groups. Differences between groups were considered statistically significant at ^*^P<0.05, ^**^P<0.01, ^***^P<0.001, and ^****^P<0.0001.

## Results

### lactate promotes M2 polarization of macrophages *in vitro*

**Figure 1:**
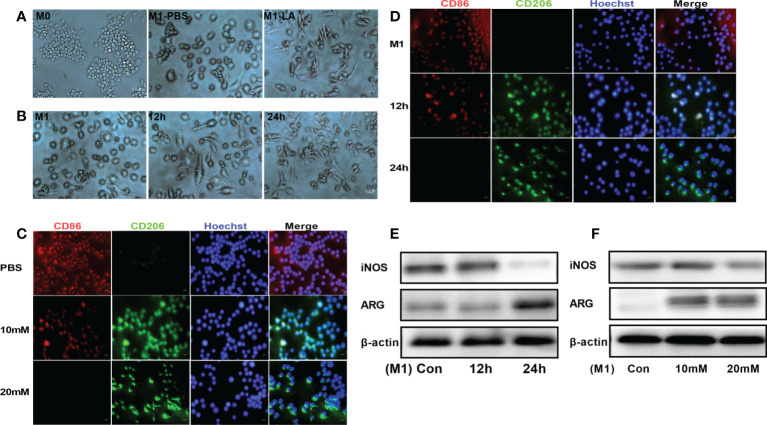
Lactate promoted M2 polarization of macrophages in vitro. **(A)** Representative images of macrophage phenotypes after lactate treatment. Polarization of macrophages toward the M2 phenotype was associated with elongated cell shape (n = 3). scale bar, 5μm. **(B)** Morphological changes following the phenotypic transformation of macrophages at different time points (n = 3). scale bar, 5μm. **(C)** Representative fluorescence images showing the macrophage phenotypes after lactate treatment (n = 3). scale bar, 5μm. **(D)** Representative fluorescence images of macrophage phenotypes transformation after lactate treatment at different time points (n = 3). scale bar, 5μm. **(E)** Western blotting assay showing the expression of phenotype-related markers in LPS-stimulated macrophages after lactate treatment at different time points (n = 3). **(F)** Expression level of M1 macrophages-related markers after a 24-h treatment with different concentrations of lactate as determined by western blotting (n = 3).

To explore the *in vitro* effects of lactate on macrophage polarization, RAW264.7 macrophages were first treated with LPS to induce inflammation and then cultured with low and high lactate concentrations. The results revealed that the M1 and M2 phenotypes of macrophages showed significantly different cell morphologies, consistent with a previous study (17). The M0 macrophages were small, round, and with a few short protrusions. Stimulation with LPS for 24 hours caused the macrophages (M1) to change morphology into large, irregular, flat-shaped cells with longer pseudopodia. The addition of lactate induced polarization of the M1 macrophages to the M2 phenotype, which showed cell elongation (**Figure 1A**). The macrophages were said to have cell elongationwhen the length of the long axis was more than twice the length of the short axis. A few elongated cells were seen at 12h. However, more cells were elongated at 24h (**Figure 1B**). We then examined the phenotypic distribution of M1 and M2 macrophages in each group. The immunofluorescence staining showed increased expression of CD86 (red fluorescence) in M1 macrophages. However, the addition of lactate decreased the expression of CD86 and increased the expression of the M2 marker CD206 (green fluorescence) (**Figure 1C**). The expression of CD206 showed a gradual increase upon incubation for longer periods. However, the expression of CD86 showed a gradual decrease upon incubation for longer periods (**Figure 1D**). In addition, Western blotting analysis showed that lactate decreased the expression of iNOS (M1 macrophage marker), and significantly increased the expression of argininase-1 (Arg1) (M2 macrophage markers). The expression of iNOS and argininase-1 was time and concentration-dependent (**Figures 1E, F**). These results showed that lactate promotes polarization of macrophages into the M2 phenotype.

### Lactate inhibits inflammation *in vitro*

**Figure 2:**
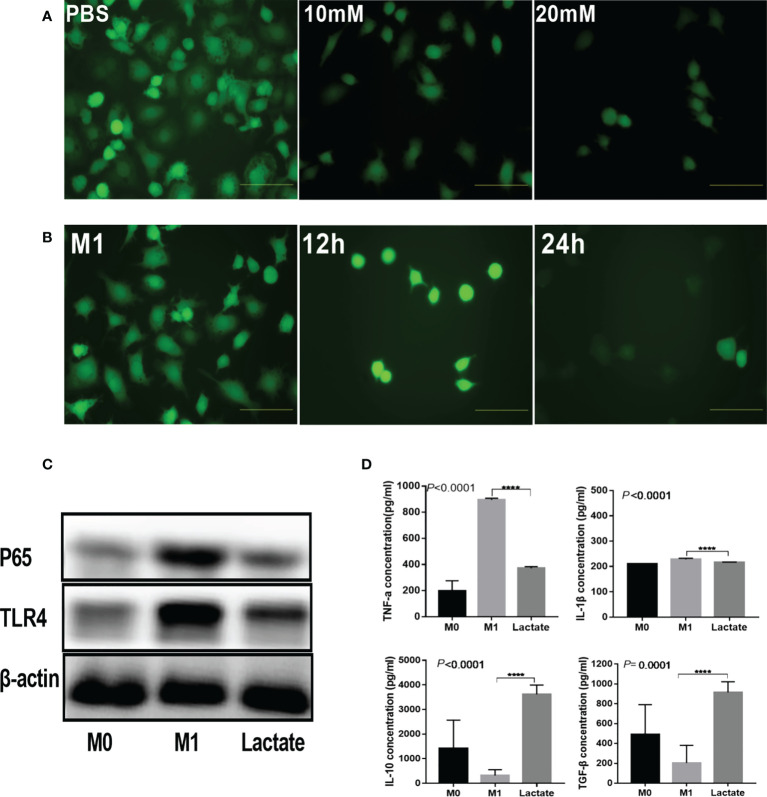
Lactate inhibited inflammation in vitro. **(A)** Intracellular ROS generation was observed using a ROS fluorescence probe DCFH-DA after lactate treatment (n = 3). scale bar, 5μm. **(B)** Intracellular ROS generation by macrophages at different time points (n = 3). scale bar, 5μm. **(C)** Western blotting results showing expression of phenotypic markers of LPS-stimulated macrophages after lactate treatment (n = 3). **(D)** Inflammatory cytokines released by macrophages in different treatment groups determined by ELISA (n=6). Pro-inflammatory cytokines, TNF-α and IL-1β; Anti-inflammatory factors, TGF-β and IL-10.

To investigate the *in vitro* effects of lactate on inflammation, we detected the inhibition of lactate on reactive oxygen species (ROS), inflammatory signaling proteins, and inflammatory factors produced by activated macrophages. The results revealed that activated M1 macrophages had significantly increased levels of ROS. However, upon incubation with lactate, the macrophages showed a significant decrease in ROS (**Figure 2A**). In addition, incubation with lactate resulted in a gradual decline in ROS levels (**Figure 2B**). These results indicated that lactate effectively inhibited ROS in M1 macrophages. The PBS group showed increased expression of the TLR4/NF-κB signaling molecules. However, treatment with lactate led to decreased expression of TLR4 and NF-κBP65 protein (**Figure 2C**). The levels of pro-inflammatory and anti-inflammatory factors in the cell-free supernatant were determined by enzyme linked immunosorbent assay (ELISA) (**Figure 2D**). The results showed that LPS induced the expression of TNF-α and interleukin 1β (IL-1β). However, incubation with lactate for 24 hours decreased the expression of pro-inflammatory mediators (TNF-α and IL-1β) and increased the expression of anti-inflammatory factors (TGF-β and IL-10). These results suggest that lactatesuppresses the pro-inflammatory response of M1 macrophages.

### Lactate has protective effects on the intestinal mucosa

**Figure 3:**
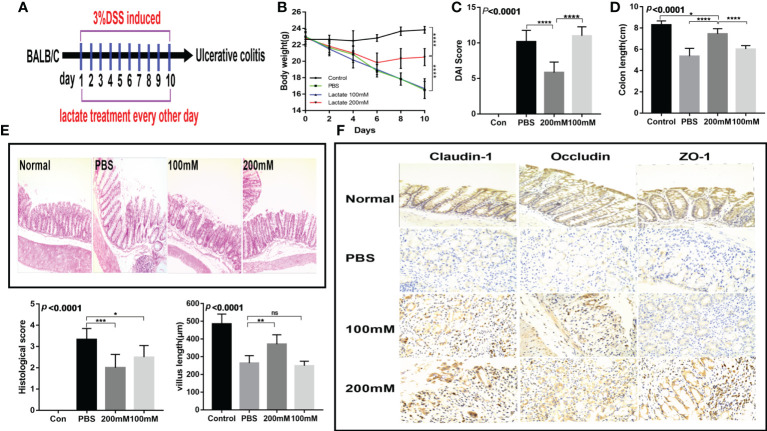
Lactate improved intestinal injury and intestinal mucosal barrier in mice with colitis. **(A)** Therapeutic design of drug-induced acute colitis model mice (n = 6). **(B)** Body weight changes in mice after lactate treatment (n = 6). **(C)** Changes of disease activity index in mice after lactate treatment (n = 6). **(D)** Colon length of mice after different treatments (n = 6). **(E)** Representative images of HE staining of colon tissues and histological scores in different groups (n = 6). Scale bar, 100μm. The length of intestinal villi of mice after different treatments (n = 6). **(F)** Immunohistochemistry images showing the level of tight junction proteins in colon tissues in different groups (n = 6). Scale bar, 100μm.

To investigate the *in vivo* effects of lactate, we established a dextran sodium sulfate (DSS) acute colitis mouse model. The mice were provided with drinking water containing lactate on days 2, 4, 6, 8, and 10 as previously described (**Figure 3A**). The results showed that the high-dose lactate group had the fastest weight recovery (**Figure 3B**) and the lowest disease activity index score (**Figure 3C**). The high-dose lactate group showed a significant improvement in colon length compared with the PBS group (**Figure 3D**). However, there was no significant difference in colon length between the low-dose and PBS groups. The histological exam revealed that the normal colon had intact villi and well-organized epithelium. In contrast, the intestinal mucosa of the PBS group showed infiltration of inflammatory cells, apparent damage to the microvilli structure, and villi atrophy. The high-dose lactate group showed reduced infiltration of inflammatory cells into the colonic tissue, preserved normal crypts and intestinal gland structure, and slightly damaged villi. In addition, lactate-treated mice showed less edema, thickened intestines, and higher histological scores than those treated with PBS. The differences were more significant for the high-dose lactate group. The PBS group showed reduced villus length compared with the high-dose lactate group (**Figure 3E**). Furthermore, the high-dose lactate group showed a higher expression of tight junction proteins (claudin-1, occluding, and ZO-1) than the PBS group (**Figure 3F**). These results indicate that lactate has protective effects on the intestinal mucosal barrier.

### Lactate alleviates intestinal inflammation in the mouse model of colitis

**Figure 4:**
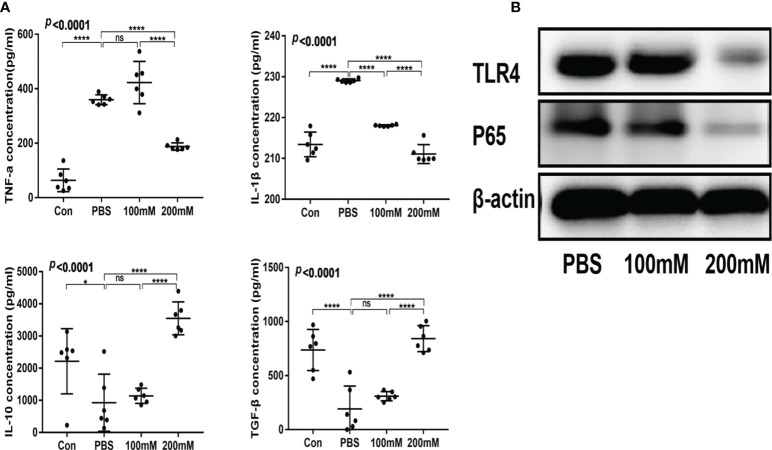
Lactic acid alleviated intestinal inflammation in mice with colitis. **(A)** Serum levels of inflammatory factors in different groups (n = 6). **(B)** Western blotting analysis of inflammatory signals TLR4 and P65 in colon tissues of different groups.

The low- and high-dose lactate groups showed reduced serum levels of TNF-α and IL-1β and increased levels of TGF-β and IL-10 compared with the PBS group (**Figure 4A**). In addition, the PBS group showed a higher expression of TLR4 and NF-κB65 proteins than the lactate groups (**Figure 4B**).

### Lactate promotes polarization of macrophages into the M2 phenotype

**Figure 5:**
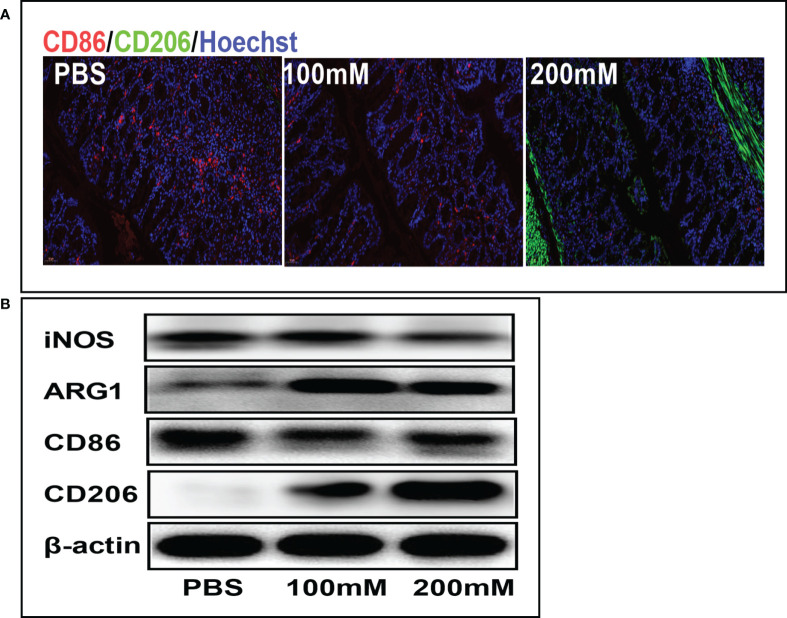
Lactic acid promoted M2 polarization of macrophages in colon tissues. **(A)** Representative fluorescent images of the macrophage phenotypes in colon tissues in different groups. Scale bar, 100μm. **(B)** Western blotting analysis showing expression level of macrophage phenotypic markers in colon tissues for different groups.

Mechanistically, lactate alleviates inflammation by promoting the phenotypic transformation of macrophages into the M2 phenotype. The immunofluorescence staining showed that the PBS group had a higher expression of CD86 (red fluorescence) than the high-dose lactate group. However, the PBS group had a lower expression of CD206 (green fluorescence) than the high-dose lactate group. These results indicate that lactate promoted the transformation of M1 macrophages (CD86+) into the M2 phenotype (CD206+) in colon tissue (**Figure 5A**). Furthermore, the Western blotting analysis showed decreased expression of CD86 and iNOS and increased expression of M2 markers CD206 and ARG-1 in the lactate group compared with the PBS group (**Figure 5B**). These results suggest that lactate reduces inflammation in colitis by polarizing macrophages into the anti-inflammatory phenotype.

### Evaluation of lactate safety

**Figure 6:**
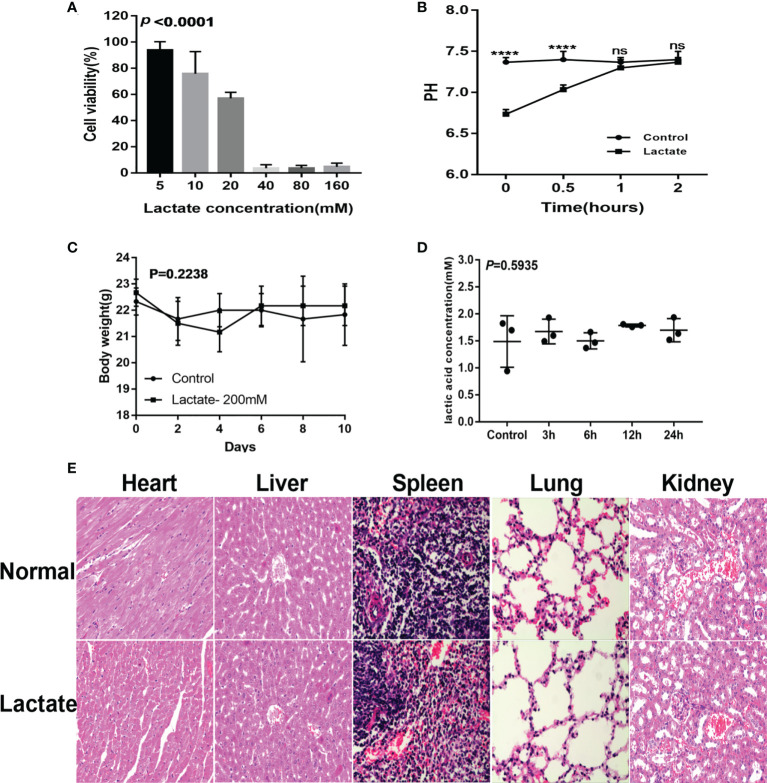
Safety evaluation of lactate. **(A)** The cytotoxicity results determined by the CCK8 assay (n=3). **(B)** Lactate was added to the cell culture media. pH was measured over 2 hours. **(C)** Effects of lactate on body weight in normal mice (N=6). **(D)** Effect of exogenous lactic acid on serum lactic acid in normal mice (n=3). **(E)** Effect of lactate on organs of normal mice (n=3).

The cell viability assay showed more than 50% cell viability demonstrating that lactate (20mM) had no apparent cytotoxic effects on macrophages (**Figure 6A**). Monitoring of the pH and buffering capacity of lactate revealed that the addition of lactate to cell cultures decreased the pH from 7.4 to 6.8. However, after 2 hours, the pH increased to 7.4 (**Figure 6B**). The *in vivo* assessments for cytotoxicity showed no significant differences in body weight between the control group and the lactate groups (**Figure 6C**). Furthermore, exogenous lactate did not affect the serum lactate concentration in mice (**Figure 6D**). Moreover, the histological examination showed no significant damage to the internal organs of mice (**Figure 6E**). These results suggest that lactate is relatively safe.

## Discussion

Dysregulation of inflammation causes various diseases, including IBD. Inflammation is a highly coordinated, complex process involving molecular, cellular, and physiological events. The resident immune cells in injured or infected tissues produce soluble mediators, including pro-inflammatory cytokines and cell adhesion molecules, which promote leukocyte migration into the inflammatory site. Excessive activation of inflammation can cause tissue damage (18). Common anti-inflammatory drugs, such as glucocorticoids, non-steroidal anti-inflammatory drugs (NSAIDs), and anti-cytokine biologics, inhibit or antagonize the effects of the inflammatory mediators and are currently the mainstay treatment for inflammatory diseases (19). However, these drugs are associated with long-term side effects. Therefore, there is a need to investigate the opportunities and challenges of translational research in inflammatory diseases.

Previous studies have shown that lactate has unique biological activities. In addition, lactate signaling inhibits inflammation. However, the mechanism is unclear. According to another review article (6), the possible mechanisms underlying the anti-inflammatory effects of lactate include (i) the anti-inflammatory effects of lactate are mediated through the lactate receptor GPR81; (ii) lactate drives a negative feedback loop to inhibit glycolysis, thus inhibiting the release of pro-inflammatory mediators by macrophages. (iii) lactate can promote steady-state macrophage polarization by inhibiting RLR signal transduction. (iv) Epigenetic modifications of histone lactylation in macrophages induce gene expression. These findings support that lactate could be exploited as a new therapeutic strategy to alleviate inflammation. This study demonstrated that lactate induces polarization of macrophages. In addition, this study revealed that lactate inhibits metabolic reprogramming in macrophages in the immune microenvironment and alleviates inflammation. The *in vitro* experiment showed that lactate inhibited the expression of TNF-α and IL-6 in LPS-stimulated macrophages, consistent with a previous study (20). Hoque et al. reported that lactate-induced GPR81 expression in leukocytes inhibited the activation of NF-κB and the inflammasome (10). The present study showed that 15-20mM lactate promoted the polarization of macrophages and inhibited inflammation, demonstrated by the decreased expression ofpro-inflammatory factors (TNF-α and IL-1β), and the increased expression of anti-inflammatory factors (TGF-β and IL-10). Carolina Iraporda et al. (21) reported that lactate showed protective effects on the TNBS-induced colitis model. They reported that lactate could alleviate histopathological damage, prevent bacterial translocation, and decrease the serum IL-6 levels, consistent with the present study.

Acidity and the lactate molecules have been shown to have similar effects on cytokine

production (22). Caslin et al. showed that sodium lactate levels ≥20 mM significantly suppressed cytokine production with no effects on viability. Once lactate and H+ ions are released from the cell and into the bloodstream, buffering systems such as bicarbonate kick in to help prevent acidosis. The present study revealed that upon the addition of lactate to the cell cultures, the pH decreased from 7.4 to 6.8. However, the pH returned to 7.4 within 2 hours and was not different from that of the negative control.

Lactate demonstrated a protective effect on the colon mucosal barrier. In addition, lactate increased the expression of tight junction proteins in the colon mucosa, thus alleviating intestinal inflammation and protecting against intestinal tissue damage. Zhang et al.reported that lactate promoted histone lactylation in macrophages, inhibited activation of macrophages and promoted polarization of macrophages to an anti-inflammatory reparative phenotype (15). The findings of the present study offer valuable insights into the mechanisms of inflammation and possible therapeutic targets to reduce inflammation.

The cytotoxicity assays showed that lactate was relatively safe. However, the clinical applicability of lactate for inflammatory diseases remains challenging because i) oral administration of lactate is not feasible as the liver has a high capacity to remove lactate from the circulation. ii) lactic acid is acidic. Therefore, future studies should explore safe, efficacious alternatives that can achieve therapeutic concentrations at the target inflammatory sites.

## Conclusion

Lactate decreases the expression of TLR4/NF-kappa B signaling proteins. In addition, lactate decreases the expression of inflammatory cytokines in activated macrophages by promoting polarization of macrophages. Furthermore, lactate promotes the repair of the intestinal mucosal barrier and protects the intestinal tissue in inflammation. Moreover, lactate has minimal side effects. Therefore, lactate is a promising drug for reducing inflammation.

## Data availability statement

The original contributions presented in the study are included in the article/supplementary materials. Further inquiries can be directed to the corresponding author.

## Ethics statement

The animal study was reviewed and approved by Animal Protection Committee of the Logistic Support Force’s 940th Hospital. Written informed consent was obtained from the owners for the participation of their animals in this study.

## Author contributions

H-CZ drafted the paper and funding acquisition. H-BL conceived, designed and revised the paper. H-CZ, X-YY and X-QL revised and finalized the paper. H-CZ and W-WY completed experiment and data curation. X-FM and J-PL completed formal analysis. X-YD and H-YM completed Methodology. All authors contributed to the article and approved the submitted version.

## Funding

This study was sustained by the Youth Science and Technology Fund Program of Gansu Province (No. 21JR11RA175) and National Natural Science Foundation of China (No. 21772080).

## Conflict of interest

The authors declare that the research was conducted in the absence of any commercial or financial relationships that could be construed as a potential conflict of interest.

## Publisher’s note

All claims expressed in this article are solely those of the authors and do not necessarily represent those of their affiliated organizations, or those of the publisher, the editors and the reviewers. Any product that may be evaluated in this article, or claim that may be made by its manufacturer, is not guaranteed or endorsed by the publisher.
